# Early Introduction and Rise of the Omicron Severe Acute Respiratory Syndrome Coronavirus 2 (SARS-CoV-2) Variant in Highly Vaccinated University Populations

**DOI:** 10.1093/cid/ciac413

**Published:** 2022-07-01

**Authors:** Brittany A Petros, Jacquelyn Turcinovic, Nicole L Welch, Laura F White, Eric D Kolaczyk, Matthew R Bauer, Michael Cleary, Sabrina T Dobbins, Lynn Doucette-Stamm, Mitch Gore, Parvathy Nair, Tien G Nguyen, Scott Rose, Bradford P Taylor, Daniel Tsang, Erik Wendlandt, Michele Hope, Judy T Platt, Karen R Jacobson, Tara Bouton, Seyho Yune, Jared R Auclair, Lena Landaverde, Catherine M Klapperich, Davidson H Hamer, William P Hanage, Bronwyn L MacInnis, Pardis C Sabeti, John H Connor, Michael Springer

**Affiliations:** Department of Systems Biology, Harvard Medical School, Boston, Massachusetts, USA; Broad Institute of Massachusetts Institute of Technology and Harvard, Cambridge, Massachusetts, USA; Division of Health Sciences and Technology, Harvard Medical School and Massachusetts Institute of Technology, Cambridge, Massachusetts, USA; Harvard/Massachusetts Institute of Technology, MD-PhD Program, Boston, Massachusetts, USA; National Emerging Infectious Diseases Laboratories, Boston, Massachusetts, USA; Bioinformatics Program, Boston University, Boston, Massachusetts, USA; Broad Institute of Massachusetts Institute of Technology and Harvard, Cambridge, Massachusetts, USA; Harvard Program in Virology, Division of Medical Sciences, Harvard Medical School, Boston, Massachusetts, USA; Department of Biostatistics, School of Public Health, Boston University, Boston, Massachusetts, USA; Department of Mathematics & Statistics, Boston University, Boston, Massachusetts, USA; Rafik B. Hariri Institute for Computing and Computational Science and Engineering, Boston University, Boston, Massachusetts, USA; Broad Institute of Massachusetts Institute of Technology and Harvard, Cambridge, Massachusetts, USA; Harvard Program in Biological and Biomedical Sciences, Division of Medical Sciences, Harvard Medical School, Boston, Massachusetts, USA; Harvard University Clinical Laboratory, Harvard University, Cambridge, Massachusetts, USA; Broad Institute of Massachusetts Institute of Technology and Harvard, Cambridge, Massachusetts, USA; Boston University Clinical Testing Laboratory, Boston University Boston, Massachusetts, USA; Integrated DNA Technologies, Inc, Coralville, Iowa, USA; Howard Hughes Medical Institute, Chevy Chase, Maryland, USA; Broad Institute of Massachusetts Institute of Technology and Harvard, Cambridge, Massachusetts, USA; Integrated DNA Technologies, Inc, Coralville, Iowa, USA; Center for Communicable Disease Dynamics, Department of Epidemiology, Harvard T.H. Chan School of Public Health, Boston, Massachusetts, USA; Integrated DNA Technologies, Inc, Coralville, Iowa, USA; Integrated DNA Technologies, Inc, Coralville, Iowa, USA; Harvard University Clinical Laboratory, Harvard University, Cambridge, Massachusetts, USA; Boston University Student Health Services, Boston, Massachusetts, USA; Section of Infectious Diseases, Boston University School of Medicine and Boston Medical Center, Boston, Massachusetts, USA; Section of Infectious Diseases, Boston University School of Medicine and Boston Medical Center, Boston, Massachusetts, USA; Student Affairs, Northeastern University, Boston, Massachusetts, USA; Department of Chemistry and Chemical Biology, Northeastern University, Boston, Massachusetts, USA; Life Sciences Testing Center, Northeastern University, Burlington, Massachusetts, USA; Biopharmaceutical Analysis and Training Laboratory, Burlington, Massachusetts, USA; Boston University Clinical Testing Laboratory, Boston University Boston, Massachusetts, USA; Department of Biomedical Engineering, Boston University, Boston, Massachusetts, USA; Boston University Clinical Testing Laboratory, Boston University Boston, Massachusetts, USA; Boston University Student Health Services, Boston, Massachusetts, USA; Boston University Precision Diagnostics Center, Boston University, Boston, Massachusetts, USA; National Emerging Infectious Diseases Laboratories, Boston, Massachusetts, USA; Section of Infectious Diseases, Boston University School of Medicine and Boston Medical Center, Boston, Massachusetts, USA; Boston University Precision Diagnostics Center, Boston University, Boston, Massachusetts, USA; Department of Global Health, Boston University School of Public Health, Boston, Massachusetts, USA; Center for Emerging Infectious Disease Research and Policy, Boston University, Boston, Massachusetts, USA; Center for Communicable Disease Dynamics, Department of Epidemiology, Harvard T.H. Chan School of Public Health, Boston, Massachusetts, USA; Broad Institute of Massachusetts Institute of Technology and Harvard, Cambridge, Massachusetts, USA; Broad Institute of Massachusetts Institute of Technology and Harvard, Cambridge, Massachusetts, USA; Howard Hughes Medical Institute, Chevy Chase, Maryland, USA; Department of Organismic and Evolutionary Biology, Harvard University, Cambridge, Massachusetts, USA; Department of Immunology and Infectious Diseases, Harvard T.H. Chan School of Public Health, Harvard University, Boston, Massachusetts, USA; Department of Medicine, Division of Infectious Diseases, Massachusetts General Hospital, Boston, Massachusetts, USA; Massachusetts Consortium on Pathogen Readiness, Boston, Massachusetts, USA; National Emerging Infectious Diseases Laboratories, Boston, Massachusetts, USA; Bioinformatics Program, Boston University, Boston, Massachusetts, USA; Department of Microbiology, Boston University School of Medicine, Boston, Massachusetts, USA; Department of Systems Biology, Harvard Medical School, Boston, Massachusetts, USA; Broad Institute of Massachusetts Institute of Technology and Harvard, Cambridge, Massachusetts, USA

**Keywords:** SARS-CoV-2, epidemiology, infectious disease surveillance

## Abstract

**Background:**

The Omicron variant of severe acute respiratory syndrome coronavirus 2 (SARS-CoV-2) is highly transmissible in vaccinated and unvaccinated populations. The dynamics that govern its establishment and propensity toward fixation (reaching 100% frequency in the SARS-CoV-2 population) in communities remain unknown. Here, we describe the dynamics of Omicron at 3 institutions of higher education (IHEs) in the greater Boston area.

**Methods:**

We use diagnostic and variant-specifying molecular assays and epidemiological analytical approaches to describe the rapid dominance of Omicron following its introduction into 3 IHEs with asymptomatic surveillance programs.

**Results:**

We show that the establishment of Omicron at IHEs precedes that of the state and region and that the time to fixation is shorter at IHEs (9.5–12.5 days) than in the state (14.8 days) or region. We show that the trajectory of Omicron fixation among university employees resembles that of students, with a 2- to 3-day delay. Finally, we compare cycle threshold values in Omicron vs Delta variant cases on college campuses and identify lower viral loads among college affiliates who harbor Omicron infections.

**Conclusions:**

We document the rapid takeover of the Omicron variant at IHEs, reaching near-fixation within the span of 9.5–12.5 days despite lower viral loads, on average, than the previously dominant Delta variant. These findings highlight the transmissibility of Omicron, its propensity to rapidly dominate small populations, and the ability of robust asymptomatic surveillance programs to offer early insights into the dynamics of pathogen arrival and spread.

## Teaser

Omicron established and fixated at universities before the surrounding state and region. Fixation was rapid despite lower viral loads in Omicron vs Delta samples. We highlight the ability of asymptomatic surveillance programs to offer early insights into pathogen dynamics.

In the final days of 2021, the global severe acute respiratory syndrome coronavirus 2 (SARS-CoV-2) case count surpassed 1 million cases per day [1], with the surge due, at least in part, to the Omicron variant of concern (B.1.1.529). In the United States, coronavirus disease 2019 (COVID-19) case counts reached record highs (3–5 times the peak of prior waves), with the estimated percentage of cases due to Omicron rapidly increasing from <1% of cases (4 December 2021) to >95% of cases (1 January 2022) [2]. Omicron transmission is possible among both vaccinated and unvaccinated individuals [3], although the relative rates of transmission from each remain unclear. Evidence suggests that Omicron can partially evade immunity acquired from prior COVID-19 infection [4] and from a 2-dose messenger RNA (mRNA) vaccine regimen [5], though a third dose improves Omicron neutralization efficiency, at least in the short term [6].

To mitigate the risks of congregate living, institutes of higher education (IHEs) use a combination of vaccination requirements [7, 8], high-frequency testing [8, 9], and behavioral interventions such as masking and social distancing to control viral spread. An analysis [10] suggests that in the setting of masking and frequent testing, case counts are not correlated with dorm occupancy or in-person instruction; this is consistent with the evidence that cases have been predominantly acquired in off-campus settings [11]. Moreover, detailed genomic analyses of an IHE and its nearby communities suggested that transmission dynamics within the IHE did not result in spread to the greater community [12]. Thus, many IHEs successfully controlled the spread of COVID-19 through the Delta surge. However, in December 2021, COVID-19 case counts rose rapidly both in college communities [13] and in New England (NE) as a whole, with viral genomic sequencing confirming Omicron as the cause. While some institutions responded by converting to distance learning or requiring booster shots [14–18], the feasibility of maintaining residential college life without another spike in cases was in question.

Here, we capitalize on asymptomatic testing programs at 3 Boston-based IHEs, Boston University (BU), Harvard University (HU), and Northeastern University (NU), to document the rapid takeover of the Omicron variant, reaching near-fixation within the span of 9.5–12.5 days despite lower viral loads, on average, than the previously dominant Delta (B.1.627.2) variant.

## METHODS

### Patient Samples and Ethics Statement

We gathered de-identified sample information from 3 institutions with campus testing programs [11] (Table 1). We received the following information for every positive test collected between 2 December 2021 and 21 December 2021: sample collection date, cycle threshold (Ct) for 1 or more genes, and variant designation. From BU, we also received affiliate status (student vs employee, where employees include faculty, staff, and contractual employees). For HU, SARS-CoV-2 samples were collected from consented individuals under the Harvard Longwood Campus institutional review board (IRB) 20-1877 and covered by an exempt determination (EX-7295) at the Broad Institute. For BU, SARS-CoV-2 samples and data access were covered by an exemption determination under BU IRB 6122E. The use of these data in this study was evaluated and approved by NU under data use agreement 20-1481.

**Table 1.** Institutions Studied

Institute of Higher Education	Individuals in Testing Program^a^	Testing Frequency^b^ (1/week)	Vaccination Rate^b^ (1 Johnson & Johnson or 2 Messenger RNA)	Variant Designation Method	Cycle Threshold Gene^c^	Cases (2–21 December 2021)	Finals Week (2021)
Boston University	43 904	1	92.5% (employees, affiliates) 97.9% (students)	Viral sequencing	N1 N2 RNase P	524	14–18 December
Harvard University	38 434	3 (live on campus) 2 (live off campus, not vaccinated) 1 (live off campus, vaccinated)	97% (employees) 98% (students)	mCARMEN variant-specific polymerase chain reaction	N1	635	9–18 December
Northeastern University	30 602	1	97.7% (employees) 99.6% (students)	S-gene target failure	N1 ORF1ab S MS2	447	10–18 December

Individuals in the testing programs include undergraduate and graduate students, faculty, and staff.

Testing frequencies and vaccination rates were collected from publicly available university dashboards [19–21].

N1, N2, ORF1ab, and S are severe acute respiratory syndrome coronavirus 2 genes; RNase P is a human gene (control); MS2 is a bacteriophage (control).

### Experimental Methods

Across universities, individuals self-collected anterior nares specimens, which were analyzed using reverse transcription quantitative polymerase chain reaction (RT-qPCR). At BU, a 2-target SARS-CoV-2 assay with RNase P control was performed [11, 22]. At HU, the Quaeris SARS-CoV-2 assay was performed [23]. At NU, the Thermo Fisher Scientific Applied Biosystems TaqPath COVID-19 Combo Kit was used [24]. Variant status was assessed using amplicon-based viral sequencing, as previously described [25] (BU); mCARMEN [26], a CRISPR-based diagnostic platform that identifies unique combinations of Spike gene mutations (HU); a variant-specific PCR assay (HU; Supplementary Table 1); or S-gene target failure [27] (SGTF; NU; Supplementary Methods).

### Data Curation

We downloaded Massachusetts (MA) and NE case count data from the Centers for Disease Control and Prevention [28] and sequence data from the Global Initiative on Sharing All Influenza Data (GISAID) [29–31]. We removed 152 of 1758 samples (8.6%) from the universities (50 from BU and 102 from HU) with missing variant information (ie, due to assay technical limitations) from all subsequent analyses. We removed 53 of the 22 211 (0.2%) MA sequences from GISAID that had a variant classification other than Delta or Omicron. We removed 22 211 of the 30 796 (72.1%) NE sequences from GISAID [29–31] that were in MA (ie, NE curve fits do not include MA) and 29 of the remaining 8585 (0.3%) sequences that had a variant classification other than Delta or Omicron. We removed 20 gene-specific data points with Ct >40 or Ct <5 due to possible technical errors. For Ct comparisons, samples with missing data due to failed amplification of a specific gene were removed solely from the analysis of that gene. For the per-affiliation analyses, we removed 6 of 524 (1.1%) BU cases with missing student or employee designations.

### Logistic Regression and Inference

We fit logistic models on binary variant calls as a function of the date, estimating the proportion of cases that were Omicron over time for each university individually (with data from 2 December–21 December), for MA and NE (with data from 1 December–1 January) and for BU by affiliation (student vs employee; with data from 2 December–21 December). We documented 95% confidence intervals (CIs) for our model’s parameters, the overdispersion ratio, and McFadden’s pseudo-R^2^ (Supplementary Methods).

We estimated the date at which the Omicron fraction reached 10%, 50%, and 90%, hereafter defined as O_10_, O_50_, and O_90_. We used the notation ΔO_x, A–B_ = O_x, Population A_ – O_x, Population B_ to represent the difference in the date at which the Omicron fraction reached x% between 2 populations, and we used the notation ΔO_90–10_ = O_90−_ O_10_ to represent the number of days it took a particular population’s Omicron fraction to rise from 10% to 90%.

We derived point estimates for O_x_ by inverting our regression model, such that:

$$O_{x}\;=\frac{logit(\frac{x}{100})-B_{0}}{B_{1}},$$

where *B*_0_ is the intercept and *B*_1_ is the slope. To generate a standard error for *O*_x_, we used the delta method [32, 33] with the transformation function *O*_x_(x) (as above) and with the mean and covariance of x determined by the coefficients and covariance matrix of our regression model, respectively.

We generated 95% CIs for *O*_x_ and compared *O*_x_ values between populations by approximating the distribution of *O*_x_ via the family of Student *t* distributions (Supplementary Methods).

### Case Counts

For MA and NE, we summed confirmed and probable daily cases into the metric total daily cases. We noted weekly variation in case reporting (ie, no MA cases were reported on the weekends; Supplementary Figure 1) and thus calculated 7-day rolling averages. We noted smaller-scale variation in case count reporting at IHEs and calculated 3-day rolling averages. To approximate the number of MA cases that were Delta or Omicron, we used our logistic regression model to estimate the Omicron fraction for each day in 1 December–15 January. We scaled case counts by population sizes provided in Table 1 (IHEs) or in the 2020 census (MA) [34].

### Ct Value Comparisons

We compared Ct values for Delta and Omicron cases per institution and per target, as each university had a unique testing protocol. We compared Ct values for the N1 (BU, HU), N2 (BU, NU), and ORF1ab (NU) genes. We also compared Ct values at BU per affiliation. We used the Wilcoxon rank sum test with Benjamini-Hochberg correction [35] to assess the relationship between SARS-CoV-2 variant and Ct.

## RESULTS

**Figure 1.**
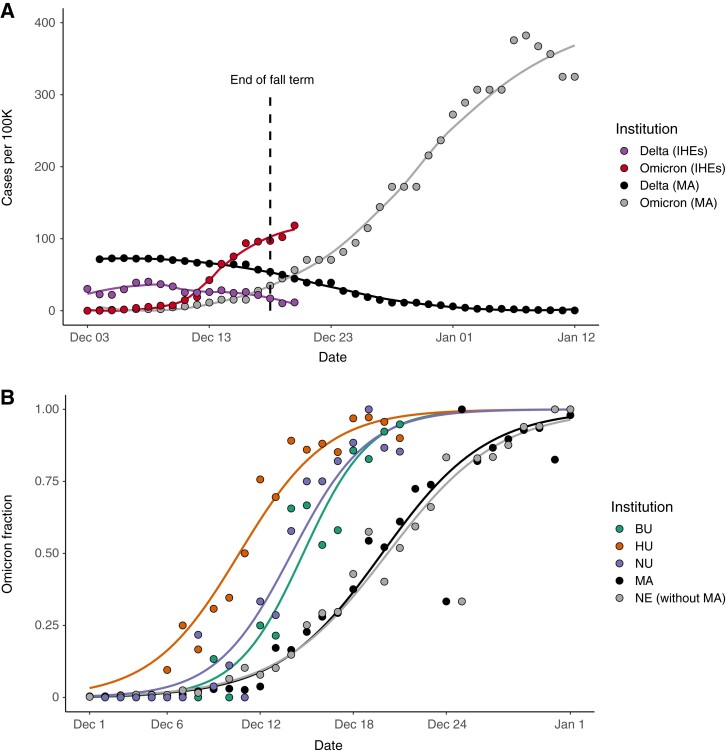
*A*, Cases per 100 000 across the IHEs and MA, stratified by variant. Plotted values are rolling averages over a 3-day (IHEs) or 7-day (MA) window to account for weekly variation in case reporting. Omicron and Delta variant proportions in MA were inferred from Global Initiative on Sharing All Influenza Data (GISAID) data (see the Methods section). The last day of fall semester finals occurred on 18 December (dashed line). Data from 3 December–20 December (IHEs) and 4 December 4–12 January (MA). *B*, Proportion of cases that were Omicron from 2 December–21 December (IHEs) and 1 December–1 January (MA, NE). Data were modeled using logistic regression. MA, Massachusetts data from GISAID. NE without MA, New England data (excluding the MA data) from GISAID. Abbreviations: BU, Boston University; HU, Harvard University; IHE, institutes of higher education; MA, Massachusetts; NE, New England; NU, Northeastern University.

There was a rapid increase in both daily case count and the Omicron fraction at IHEs in December 2021. In early December, Delta was circulating across MA and at IHEs, though case rates were higher per capita in the community outside of IHEs (Figure 1A). The Omicron surge at IHEs in mid-December was accompanied by a more modest rise in case counts (ie, slope of cases vs time) in MA and NE during the same period (note: testing rates were lower in these populations), followed by a striking regional surge in late December (Figure 1A, Supplementary Figure 1). A total of 1606 SARS-CoV-2 cases were identified across the 3 institutions (BU, HU, and NU) between 2 December (0% Omicron) and 21 December (91% Omicron). The fraction of cases that were Omicron across the IHEs, MA, and NE displayed a classic sigmoid-shaped curve consistent with logistic growth (Figure 1B, Supplementary Table 2), moving toward Omicron fixation. Delta diminished in frequency as well as total case count. By 5 January, the Harvard University Clinical Laboratory found that 100% of 159 samples tested were Omicron.

Omicron was established earlier and rose to fixation faster at IHEs than in MA as a whole (Figure 1B). We noted that MA and NE (without MA) had visually indistinguishable curves and fitted parameters with highly overlapping CIs (Supplementary Table 2). Thus, we compared the timing of Omicron’s trajectory between IHEs and MA, with results generalizable to NE. To compare the timing of Omicron establishment across populations, we generated a metric (O_10_; see the Methods section) that estimates the date range at which 10% of the cases were Omicron. O_10_ occurred significantly earlier at IHEs than in MA, by an average of 2.4 days (BU), 3.8 days (NU), and 9.2 days (HU) (Table 2, Supplementary Table 3). To compare the duration at which Omicron fixated across populations, we generated the metric ΔO_90–10_, the duration (in days) during which Omicron rose from 10% to 90% of cases (see the Methods section). ΔO_90–10_ was 9.5 at HU (95% CI, 9.2–9.8), 10.8 at BU (95% CI, 10.4–11.1), 12.5 at NU (95% CI, 12.1–12.9), and 14.8 in MA (95% CI, 14.8–14.9), indicating that the trajectory to Omicron fixation occurred more rapidly at IHEs (Table 2). Taken together, these data point toward Omicron’s earlier establishment and faster rise to fixation at IHEs compared with MA or NE.

**Table 2.** University-Specific Point Estimates and 95% Confidence Intervals for O_x_ and for ΔO_90–10_

Institution	O_10_	O_10_ 95% CI	O_50_	O_50_ 95% CI	O_90_	O_90_ 95% CI	ΔO_90–10_ (Days)	ΔO_90–10_ 95% CI (Days)
Harvard University	4 December	2–6 December	10 December	9–11 December	16 December	15–17 December	9.5	(9.2–9.8)
Northeastern University	8 December	7–10 December	14 December	13–14 December	19 December	18–20 December	12.5	(12.1–12.9)
Boston University	10 December	8–11 December	14 December	14–15 December	19 December	18–20 December	10.8	(10.4–11.1)
Massachusetts	12 December	12 December	19 December	19–20 December	27 December	27 December	14.8	(14.8–14.9)

O_x_, the date at which the Omicron fraction equals *x* percent. ΔO_90–10_, the duration of time that it takes the Omicron fraction to rise from 10% to 90%. CIs were generated via the Student *t* distribution, with estimation of the standard errors via the delta method (see the Methods section).

Abbreviation: CI, confidence interval.

**Figure 2.**
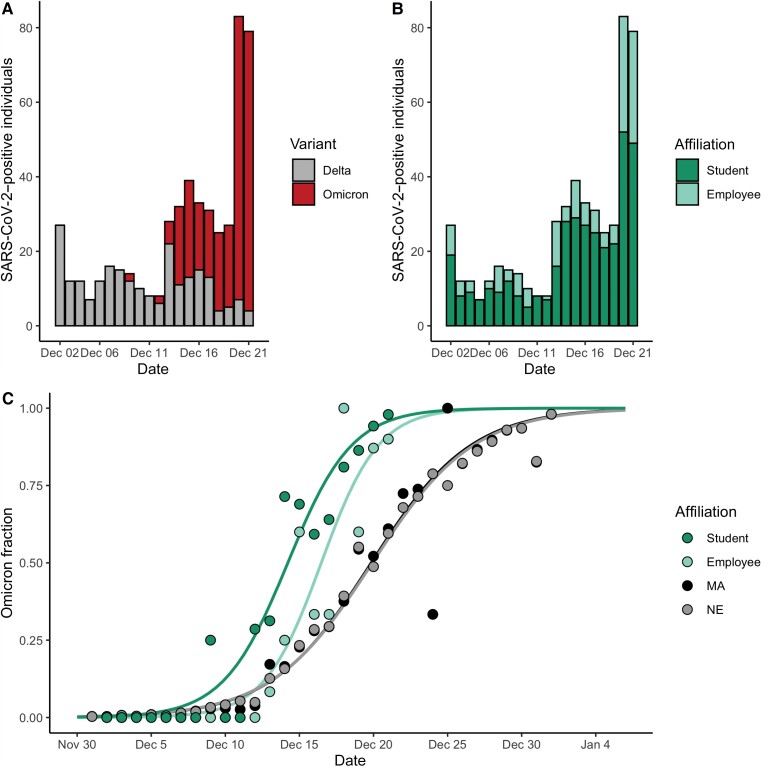
*A*, Total number of cases at Boston University (BU) stratified by variant and stacked from 2 December–21 December. Gray, Delta; red, Omicron. *B*, Total number of cases at BU stratified by affiliation status and stacked from 2 December–21 December. Light green, employees; green, students. *C*, Proportion of cases that were Omicron from 2 December–21 December (BU students and employees) and 1 December 1–1 January (MA). Data were modeled using logistic regression. Massachusetts data from Global Initiative on Sharing All Influenza Data. Abbreviations: MA, Massachusetts; NE, New England; SARS-CoV-2, severe acute respiratory syndrome coronavirus 2.

Next, we found that BU employees displayed Omicron dynamics similar to those of BU students, with a 2–3 day delay in onset. We found no significant association between affiliation (student vs employee) and variant (Figure 2A; Fisher exact test, *P* = .12, odds ratio = 0.7 with 95% CI, .5–1.1), with employees accounting for 28.7% (74 Delta, 73 Omicron) of cases (1.13 per 100 employees) and students accounting for 71.3% (157 Delta, 214 Omicron; Figure 2B) of cases (1.20 per 100 students). We again used O_10_ (see the Methods section) to compare the timing of Omicron establishment between populations. O_10_ occurred significantly earlier among BU students relative to BU employees (by an average of 2.8 days) and MA (by an average of 3.0 days; Figure 2C, Table 3, Supplementary Table 5). We compared ΔO_90–10_ as a metric of time to fixation, which was 8.5 among BU employees (95% CI, 7.9–9.1) and 9.5 among BU students (95% CI, 9.1–9.9; Table 3). ΔO_90–10_ was more comparable between BU students and employees than BU was to other IHEs (Table 2) and markedly differed in MA (14.8; 95% CI, 14.8–14.9), indicating that employees’ trajectories resembled those of students (Table 3). Taken together, we found that employees and students had parallel Omicron trajectories with a lag time between 2 and 3 days.

**Table 3.** Affiliation-Specific Point Estimates and 95% Confidence Intervals for O_x_ and for ΔO_90–10_

Affiliation	O_10_	O_10_ 95% CI	O_50_	O_50_ 95% CI	O_90_	O_90_ 95% CI	ΔO_90-10_ (days)	ΔO_90-10_ 95% CI (days)
Boston University employees	12 December	10–14 December	16 December	15–17 December	20 December	19–22 December	8.5	(7.9–9.1)
Boston University students	9 December	7–10 December	14 December	13–14 December	18 December	17–20 December	9.5	(9.1–9.9)
Massachusetts	12 December	12 December	19 December	19–20 December	27 December	27 December	14.8	(14.8–14.9)

O_x_, the date at which the Omicron fraction equals *x* percent. ΔO_90–10_, the duration of time that it takes the Omicron fraction to rise from 10% to 90%. CIs were generated via the Student *t* distribution, with estimation of the standard errors via the delta method (see the Methods section).

Abbreviation: CI, confidence interval.

**Figure 3.**
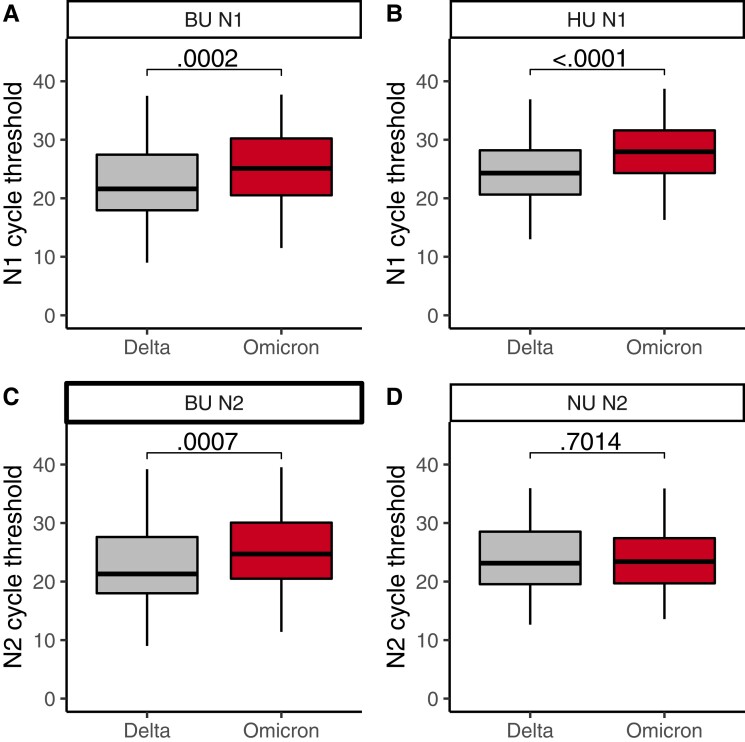
N1 cycle threshold for Delta vs Omicron cases at BU (*A*) and HU (*B*). N2 cycle threshold for Delta vs Omicron cases at BU (*C*) and NU (*D*). Gray, Delta; red, Omicron. The first, second, and third quartiles are within the box, with the median line bolded. The whisker length is 1.5 times the interquartile range (IQR), unless the furthest point is less than 1.5*(IQR) from the quartile. Outliers are displayed as points. *P* values via the Wilcoxon rank sum test and corrected via the Benjamini-Hochberg method (across the 4 comparisons in Figure 3 and the 1 comparison in Supplementary Figure 2). Abbreviations: BU, Boston University; HU, Harvard University; NU, Northeastern University.

Finally, we compared Ct values across variants and found that Omicron samples did not have lower Ct values (ie, higher viral loads) than Delta samples, suggesting that increased Omicron transmission is not driven by higher viral loads (Figure 3). At BU and HU, N1-gene Ct values were significantly higher, by an average of 2.2 (*P* = .0002) and 3.1 (*P* < .0001), respectively, in Omicron vs Delta samples (Figure 3A, 3B; Supplementary Table 6). This trend was recapitulated for N2-gene Ct values at BU (Omicron Ct values an average of 2.0 higher, *P* = .0007; Figure 3C, Supplementary Table 6). At NU, neither the N2- nor the ORF1ab-gene Ct values differed by variant (Figure 3D, Supplementary Figure 2, Supplementary Table 6). We found no difference in BU’s Ct values by affiliation status (ie, student vs employee; N1 gene, *P* = .91; N2 gene, *P* = .81), and we found that Ct-by-variant trends identified at BU are conserved when the data are conditioned on an affiliation (Supplementary Figure 3, Supplementary Table 7). In summary, we found that, despite differences in testing cadence, testing platform, and demographics among IHEs, Omicron viral Ct values were always higher than or indistinguishable from Delta Ct values.

## DISCUSSION

Here, we document Omicron’s swift spread through Boston-based IHEs in December 2021, which led to unprecedented increases in case counts. Though the IHEs and the urban environment in which they are located were experiencing Delta transmission at the time of Omicron introduction, Omicron rapidly became the dominant variant. Over an 9- to 13-day period, variant proportions converted from >90% Delta to >90% Omicron. Importantly, the rapid increase in Omicron case counts was identified in highly vaccinated populations in which Omicron’s viral load, as inferred from anterior nares diagnostic Ct, was comparable to or lower than that of Delta. This is consistent with other reports that used throat or oropharyngeal swabs [36, 37], suggesting that the difference in viral loads is not specific to the anterior nares. This highlights that Omicron’s fitness is neither driven by a higher viral load nor reliant on an immunologically naive population.

Though the date of establishment differed at the 3 IHEs, the dynamics of Omicron takeover were strikingly similar. The rapid rise in the Omicron fraction was offset by 1–4 days between universities, though we cannot rule out differences in testing cadence as the cause of the lag. The dynamics of Omicron dominance were similar across campuses despite differences in testing programs, on-campus vs off-campus housing, and variant designation technologies. Additionally, the time to fixation for BU employees was more comparable to that of BU students than that of the state, supporting a transmission mode that is independent of the residential nature of college campuses.

In contrast to the early establishment and dominance in the IHEs in our study, Omicron’s procession toward fixation in MA occurred more slowly. While the difference in introduction time could be accounted for by earlier detection of cases in asymptomatic testing programs, the differences in slope and time to fixation cannot be explained by this factor. These dynamics are consistent with overdispersion in transmission, in which clusters of cases are responsible for the majority of spread, and the early stages of establishment within a community are stochastic and scale with the number of introductions [38, 39]. Overdispersion in SARS-CoV-2 transmission is well documented [38, 40–42], and our work is consistent with the continuation of this phenomenon with Omicron. IHEs are not siloed in their interaction networks; however, the proportion of interactions within an IHE’s network is greater than the proportion of interactions that exit into the community; as a result, clusters of transmission are readily detected via robust screening. The ability of Omicron to rapidly spread through a vaccinated population means that even communities that escape the initial peak of Omicron in the United States require continued monitoring. For example, rural communities may experience late introductions of Omicron and may not notice its arrival until a significant proportion of the population has become infected. This is of particular concern in areas with low vaccination rates, where a rapid rise in case counts can overwhelm healthcare systems.

There are technical limitations to the generalizability of this study. SGTF, caused by the deletion of amino acids 69–70, was one method we used for variant designation. While SGTF occurs in multiple SARS-CoV-2 variants, the Delta variant, which lacks this deletion, was the predominant circulating variant before Omicron’s arrival. Thus, SGTF was sensitive and specific (94.6% and 99.5%, respectively, inferred from GISAID data [29–31]) for Omicron in MA during the study period. Moreover, while it is possible that differences in viral loads are confounded by differences in timing of viral incubation or clearance, longitudinal sampling of infected individuals suggests that Omicron samples have lower peak viral loads than Delta samples [36]. Finally, there are multiple potential models for the relationship between student and employee dynamics, including student–employee transmissions and transmissions that cycle between students, the community, and employees. However, we lack the viral genomic sequencing, contact tracing, and community testing data necessary to distinguish these possibilities.

Moreover, IHEs differ from MA in social structure and demographics, which may play a role in Omicron dynamics. Though universities include individuals from different communities, the age distribution, residential life, and extracurricular activities could influence Omicron dynamics. Furthermore, the degree to which potential superspreader events could be important for the initial seeding of Omicron and its continued spread is not captured here. It is possible that the spread of Omicron in non-IHEs may be slower if the structure of the social network differs [43], resulting in fewer opportunities for clustered transmission. We cannot separate possible sociobehavioral factors, such as an increase in indoor gatherings prior to the start of final examinations or in anticipation of the winter holidays, from properties intrinsic to the virus that may affect overdispersion. Finally, lower vaccination rates in MA may contribute to the relative fitness of Delta vs Omicron, and testing in MA may be biased if symptomatic testing occurs more frequently with Delta than with Omicron.

What can we learn from the spread of Omicron through universities that could help us mitigate future waves of SARS-CoV-2 or other pathogens? First, sites that have characteristics like IHEs can be informative early detection sites. We note 2 of many reasons. First, IHEs include individuals from a variety of backgrounds who intermix at the university and in the larger community, and IHEs have implemented university-wide asymptomatic screening programs. Screening programs like these can catch and categorize infections well before trends are noted in the larger community and have the potential to forecast testing needs and hospital admissions. Second, it is extremely difficult to stop the spread of a highly transmissible virus once it has become established in a community. BU, HU, and NU controlled the spread of previous variants of SARS-CoV-2 via a combination of high-cadence testing, isolation of positive individuals, contract tracing, quarantining of close contacts, social distancing, masking, vaccination requirements, and ventilation improvements. These measures were not sufficient to stop the spread of Omicron, and both BU and HU mitigated further spread via remote learning during the January term. This emphasizes the need for continued surveillance programs to rapidly identify and mitigate outbreaks before they become pandemics.

## Supplementary Data

Supplementary materials are available at *Clinical Infectious Diseases* online. Consisting of data provided by the authors to benefit the reader, the posted materials are not copyedited and are the sole responsibility of the authors, so questions or comments should be addressed to the corresponding author.

## Notes

***Author contributions*.** B. A. P. and J. T. accessed and verified the underlying data reported in the article. B. A. P. conducted statistical analyses, and L. F. W. and E. D. K. provided guidance for the analyses. B. A. P. and J. T. produced software and visualizations. J. T. collected and curated Boston University (BU) data, and L. D.-S., J. T. P., K. R. J., T. B., L. L., C. M. K., and D. H. H. provided assistance. N. L. W. collected and curated mCARMEN data with help from M. R. B., S. T. D., and T. G. N. Variant-specific polymerase chain reaction assay was collected and curated by M. C. and M. H. using custom primers and probes designed by M. S., M. G., D. T., and E. W. Project administration was aided by P. N. B. P. T. curated the Centers for Disease Control and Prevention (CDC) and Global Initiative on Sharing All Influenza Data (GISAID) data. J. R. A. and S. Y. collected and curated Northeastern University (NU) data. B. A. P. wrote the original draft of the manuscript with feedback provided by W. P. H., P. C. S., J. H. C., and M. S. The manuscript was reviewed and edited by N. L. W., P. N., K. R. J., T. B., W. P. H., P. C. S., J. H. C., and M. S. Authors W. P. H., P. C. S., J. H. C., and M. S. conceptualized and supervised the study and reviewed and edited the final manuscript.

***Acknowledgments*.** We gratefully acknowledge the students and employees at each participating university, and the staff at the BU, HU, and NU clinical testing laboratories.​​ We gratefully acknowledge the authors from the originating laboratories responsible for obtaining the specimens and the submitting laboratories where genetic sequence data were generated and shared via GISAID, on which this research is based.

***Disclaimer.*** The content is solely the responsibility of the authors and does not necessarily represent the official views of the National Institute of General Medical Sciences (NIGMS) or the National Institutes of Health (NIH).

***Data sharing.*** All submitters of data may be contacted directly (www.gisaid.org). Access to patient sample metadata was facilitated by the Massachusetts Consortium on Pathogen Readiness (MassCPR). R analyses are available at https://github.com/bpetros95/omi-uni.

***Financial support*.** This work was supported by the National Institute of General Medical Sciences grant T32GM007753 to B. A. P. and grant R35GM141821 to L. F. W.; BU for severe acute respiratory syndrome coronavirus 2 (SARS-CoV-2) surveillance to J. H. C.; MassCPR to J. H. C., W. P. H., T. P. B., and K. R. J.; the China Evergrande Group to J. H. C.; the NIH grant NIAID K23 and grant AI152930-01A1 to T. B.; the CDC SARS-CoV-2 Baseline Genomic Surveillance contract (grant 75D30121C10501 to the Broad Institute via the Clinical Research Sequencing Platform, LLC) to B. L. M.; a CDC Broad Agency Announcement grant 75D30120C09605 to B. L. M.; the Rockefeller Foundation grant 2021 HTH 013 to B. L. M. and P. C. S.; the National Institute of Allergy and Infectious Diseases grant U01AI151812 to P. C. S.; the Howard Hughes Medical Institute Investigator award to P. C. S.; and the NIH grant 5R01GM120122 to M. S. D. H. H. reports funding support for SARS-CoV-2 research from BU. C. M. K. reports support for this work from BU internal funding. D. T. reports support for this work paid to author from Integrated DNA Technologies. This work is made possible by support from Flu Lab and a cohort of donors through the Audacious Project, a collaborative funding initiative housed at TED, including The ELMA Foundation, MacKenzie Scott, the Skoll Foundation, and Open Philanthropy.

## Supplementary Material

ciac413_Supplementary_Data
