# Supplementary material for: Early Introduction and Rise of the Omicron Severe Acute Respiratory Syndrome Coronavirus 2 (SARS-CoV-2) Variant in Highly Vaccinated University Populations
Source: Clin Infect Dis. 2022 Jul 1;76(3):e400–8. doi: 10.1093/cid/ciac413 (PMC9213864; doi:10.1093/cid/ciac413)
Supplement: ciac413_Supplementary_Data [file ciac413_supplementary_data.docx]

**Supplementary Materials**

Supplemental Methods 1

- 1. Experimental Methods 1
  2. Analytic Methods 2

Supplementary Table 1 5

Supplementary Figure 1 6

Supplementary Table 2 7

Supplementary Table 3 8

Supplementary Table 4 10

Supplementary Table 5 11

Supplementary Figure 2 12

Supplementary Table 6 13

Supplementary Figure 3 14

Supplementary Table 7 15

References 16

**Supplemental Methods**

**Experimental methods:**

At BU, affiliates self-collected anterior nares specimens, which were analyzed by RT-qPCR as previously described [[1]](https://paperpile.com/c/xPLGNk/ekA2Q). Variant status was assessed using amplicon-based viral sequencing [[2]](https://paperpile.com/c/xPLGNk/FRBJn) with the ARTIC v4 primer set and the PANGOLIN lineage classification algorithm, as previously described.

At HU, affiliates self-collected anterior nares specimens, which were rehydrated with 300 μL phosphate-buffered saline (PBS) and inactivated at 65 degrees Celsius. RT-qPCR was performed using the Quaeris SARS-CoV-2 assay [[3]](https://paperpile.com/c/xPLGNk/Q4Njl), and Cts for the SARS-CoV-2 N1 and RdRP genes (and the human RNase P gene, as a positive control) were determined using the Applied Biosystems QuantStudio 7 Real-Time PCR instrument (software version 1.7).

The mCARMEN platform [[4]](https://paperpile.com/c/xPLGNk/gjHvO) distinguishes between the Delta and Omicron variants using Spike gene mutation signatures (Delta = del156/157, L452R; Omicron = del69/70, K417N, S477N, N501Y, P681H). It was run on 101 samples with 97% concordance to next-generation sequencing (NGS) and on 1,557 samples with 99.5% concordance to NGS [[4]](https://paperpile.com/c/xPLGNk/gjHvO). RNA extraction was performed using the Thermo Fisher Scientific Applied Biosystems^TM^ MagMAX^TM^ mirVana Total RNA Isolation Kit and the Spike gene was amplified using the Thermo Fisher SuperScript™ IV One-Step RT-PCR System prior to running mCARMEN.

The variant-specific PCR assay discriminates between Delta and Omicron *via* detection of the following Spike protein SNVs: L452R, Q498R, N501Y (primer and probe sequences: **Supplementary Table 1**). Primers and probes were verified on samples that were confirmed, *via* genomic sequencing, to be Delta or Omicron. Of the 384 samples tested, 343 yielded a call in 2 or 3 of the variant PCR reactions, leading to a variant designation; the 41 samples with only a single variant PCR call were excluded from the data set.

At Northeastern University, affiliates self-collected anterior nares specimens, and RNA was extracted from the clinical specimens using the Thermo Fisher Scientific Applied Biosystems^TM^ MagMAX^TM^ Viral/Pathogen II (MVP II) Nucleic Acid Isolation Kit. Cycle thresholds were determined *via* RT-qPCR, conducted with the Thermo Fisher Scientific Applied Biosystems^TM^ TaqPath^TM^ COVID-19 Combo Kit (with primers and probes specific to the N2, ORF1ab, and S genes [[5]](https://paperpile.com/c/xPLGNk/ivKEu)) according to the manufacturer’s instructions. Samples with S-gene target failure (SGTF) were designated as Omicron and samples with S-gene target amplification were designated as Delta.

**Analytic methods:**

**Software specifications:**

Analyses were run in R version 4.0.2 (2020-06-22) on a 64-bit Linux/GNU PC and on a Macbook Pro with a Darwin 17.0 platform and R version 4.1.1 (2021-08-10), with no issue reproducing analyses on each machine. Required packages include base, boot, cowplot, ggpubr, msm, reshape2, rstatix, stats, tidyverse, and zoo. Custom R scripts are available at <https://github.com/bpetros95/omi-uni>. De-identified input data is available upon request.

**Logistic regression:**

We downloaded cases counts by state over time from the CDC [[6]](https://paperpile.com/c/xPLGNk/sh04M) on January 19, 2022. We downloaded MA and NE GISAID data [[7–9]](https://paperpile.com/c/xPLGNk/fEMXW+34pJx+j505P) on January 12, 2022. We noted that MA was overrepresented in the NE data (MA contains approximately 47% of the region’s population, but 72% of available sequences), and thus removed MA from the NE data.

We fit logistic curves to the data using R’s generalized linear models package:

$Omicron fraction = \frac{e^{B0+B1*(date)}{}}{{1+ e}^{B0+B1*(date)}}$.

We calculated overdispersion ratios [[10,11]](https://paperpile.com/c/xPLGNk/zeP8M+zSjAH) to assess for the possibility of unaccounted-for variability in our models:

$Overdispersion ratio = \frac{residual deviance}{residual degrees of freedom}$.

We also calculated McFadden’s [[12,13]](https://paperpile.com/c/xPLGNk/1t9cj+gbo4t) pseudo-R^2^ as follows, where *L* is the likelihood function, the null model (“null”) regresses our data as a function of a constant (*i.e.*, 1), and the alternative model (“model”) regresses our data as a function of the date:

$1 - \frac{log(L_{model})}{log({L_{null})}}$.

The logistic growth model was a reasonable choice: (1) McFadden’s pseudo-R^2^ was in [0.31, 0.56] for all seven regression models (**Supplementary Tables 2 & 4**); and (2) there was no evidence of overdispersion, with ratios in [0.56 - 0.81] (**Supplementary Tables 2 & 4**).

We generated standard errors for O_x_ using the delta method (from R’s multi-state Markov package), and used these standard errors for inference as follows. In all cases, the sample size for each population was the number of days in our logistic regression models (*e.g.*, for BU, sample size = 20 as we included data from December 2-21):

1. We generated 95% CIs for O_x_ by assuming O_x_ approximately follows a student’s t distribution (*i.e.*, point estimate +/- *t**(standard error)), where *t* is the 97.5^th^ percentile of a student’s t distribution with degrees of freedom equal to one less than the sample size.
2. We compared O_x_ between populations – *i.e.*, ΔO_x,A-B_ = O_x, Population A_ - O_x, Population B_ – by running a student’s t-test.
3. We generated 95% CIs for ΔO_90-10_ = O_90_ - O_10_ by assuming that ΔO_90-10_ approximately follows a student’s t distribution (*i.e.*, point estimate +/- *t**(standard error)), where *t* is the 97.5^th^ percentile of a student’s t distribution with degrees of freedom equal to one less than the sample size.

**Comparisons of logistic fits:**

The logistic regression models were also compared, with the intercept parameter providing a metric for Omicron introduction time, and the slope parameter serving as a metric of the speed of fixation. The intercept parameters of our logistic regression models (B_0_) were significantly lower at BU and at NU than in MA or NE, and trends earlier at HU than in MA or NE (**Supplementary Table 2**). Moreover, the slope parameters of our logistic regression models (B_1_) were significantly steeper at BU (95% CI, 0.39-0.55) and NU (95% CI, 0.34-0.48) than in MA or NE (95% CI, 0.29-0.30), and trends steeper at HU (95% CI, 0.29-0.41) than in MA or NE (**Supplementary Table 2**). Taken together, our models provide support for earlier introductions and increased speed to fixation at IHEs.

We compared our intercepts and slopes across BU affiliations as well. The intercept parameters of our logistic regression models (B_0_) were significantly lower among both BU students and employees than in MA (**Supplementary Table 5**). Moreover, the slope parameters of our logistic regression models (B_1_) were significantly steeper among BU employees (95% CI, 0.37-0.70) and students (95% CI, 0.37-0.57) than in MA (95% CI, 0.29-0.30; **Supplementary Table 5**). These data suggest that BU employees were similar to students with respect to their trajectories to Omicron fixation.

| **SNV** | **Forward Primer** | **Reverse Primer** | **Probe** | **Quencher** |
| --- | --- | --- | --- | --- |
| L452R | CTTGATTCTA  GGTTGGTGGT  AT | CGGCCTGATA  ATTTCAGTTG | /5YakYel/TA+C+C+T+GTATA+G+A  TG/3IABkFQ/ | /56-FAM/TAC+C+G+GTA+TA+G+  AT/3IABkFQ/ |
| N501Y | CGGTAGCACA  CTTGTAATG | ACTACTACTCT  TATGGTTGGTAA | /5YakYel/CC+CAC+T+A+AT+GG+T  G/3IABkFQ/ | /56-FAM/CC+CAC+T+T+AT+GG+  TG/3IABkFQ/ |
| Q498R | CGGTAGCACA  CTTGTAATG | ACTACTACTCT  TATGGTTGGTAA | /5HEX/TT+CC+A+A+CCC+A+CT/3I  ABkFQ/ | /56-FAM/TT+CC+A+G+CCC+AC/3  IABkFQ/ |

**Supplementary Table 1.** Primer and probe sequences used to determine whether a subset of samples at Harvard University were Delta *vs.* Omicron.
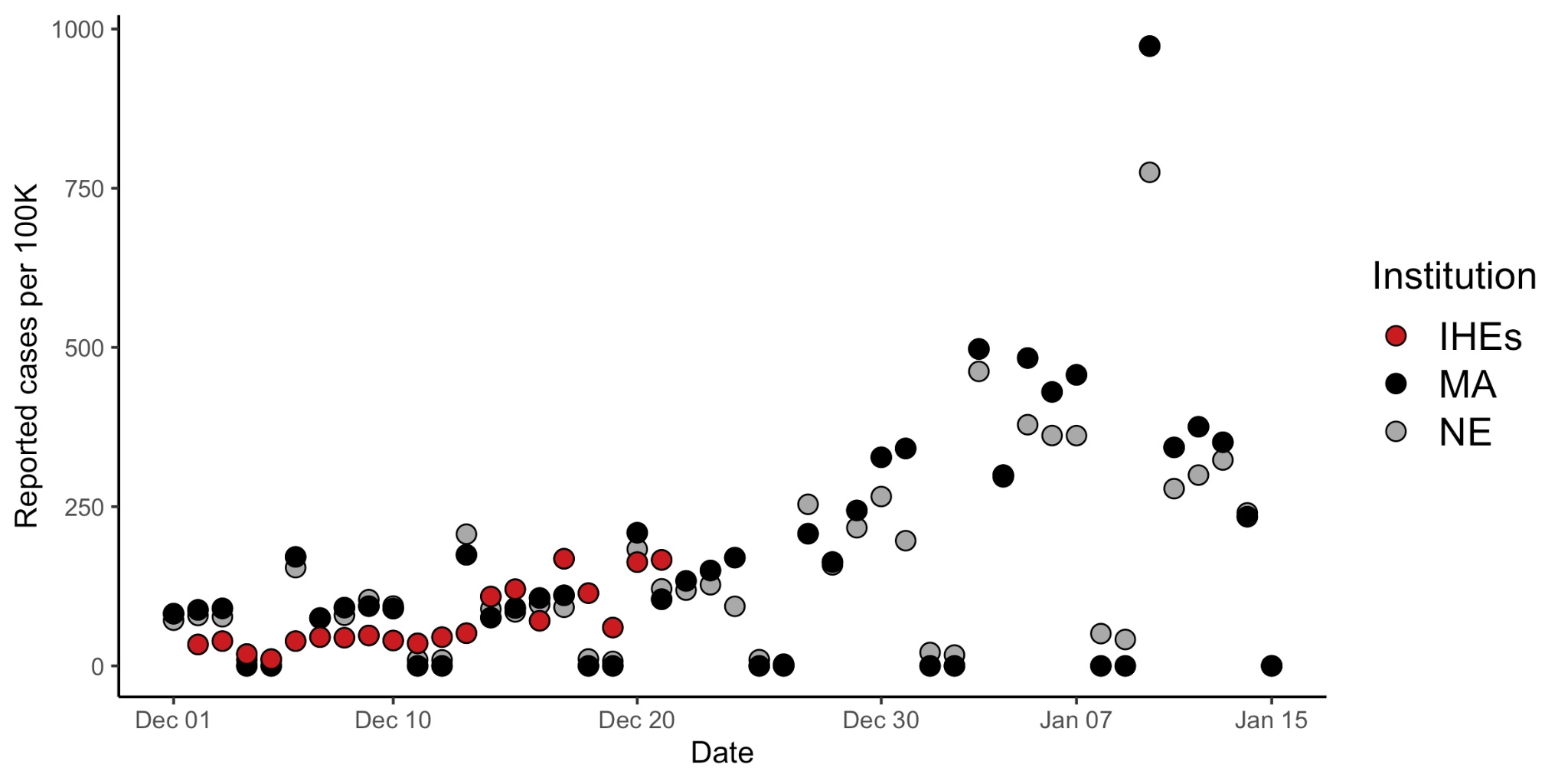


**Supplementary Figure 1.** Reported daily cases per 100,000 across the institutes of higher education (IHEs), Massachusetts (MA), and NE (New England). No methods were used to account for weekly variation in case reporting. Data from Dec 2 – 21 (IHEs) and Dec 1 – Jan 15 (MA, NE).

| **Institution** | **B_0_** | **B_0_ 95% CI** | **B_1_** | **B_1_ 95% CI** | **McFadden’s**  **R^2^** | **Overdispersion**  **ratio** |
| --- | --- | --- | --- | --- | --- | --- |
| BU | -8787.44 | (-10408.66,  -7358.17)* | 0.46 | (0.39, 0.55)* | 0.47 | 0.73 |
| HU | -6672.11 | (-7847.37,  -5595.88)* | 0.35 | (0.29, 0.41)* | 0.31 | 0.78 |
| NU | -7751.57 | (-9194.38,  -6462.90)* | 0.41 | (0.34, 0.48)* | 0.41 | 0.81 |
| MA | -5622.16 | (-5769.38,  -5478.22)* | 0.29 | (0.29, 0.30)* | 0.56 | 0.56 |
| NE | -5283.40 | (-5524.90,  -5049.55)* | 0.30 | (0.29, 0.30)* | 0.45 | 0.58 |

**Supplementary Table 2**. Logistic regression point estimates and 95% confidence intervals (CI) for intercept (B_0_) and slope (B_1_) parameters for each university. * signifies that the 95% confidence interval does not include 0. BU, Boston University. HU, Harvard University. MA, Massachusetts. NU, Northeastern University. NE, New England.

| **Institution A** | **Institution B** | **x** | **ΔO_x,A-B_ (days)** | **P-value** |
| --- | --- | --- | --- | --- |
| BU | HU | 0.1 | 5.6 | < 0.0001 |
| BU | MA | 0.1 | 2.4 | < 0.0001 |
| BU | NU | 0.1 | 1.4 | < 0.0001 |
| HU | MA | 0.1 | 8.0 | < 0.0001 |
| HU | NU | 0.1 | -4.3 | < 0.0001 |
| MA | NU | 0.1 | 3.8 | < 0.0001 |
| BU | HU | 0.5 | 4.1 | < 0.0001 |
| BU | MA | 0.5 | 5.1 | < 0.0001 |
| BU | NU | 0.5 | 0.7 | < 0.0001 |
| HU | MA | 0.5 | 9.2 | < 0.0001 |
| HU | NU | 0.5 | -3.4 | < 0.0001 |
| MA | NU | 0.5 | 5.8 | < 0.0001 |
| BU | HU | 0.9 | 2.6 | < 0.0001 |
| BU | MA | 0.9 | 7.8 | < 0.0001 |
| BU | NU | 0.9 | 0.1 | 0.44 |
| HU | MA | 0.9 | 10.4 | < 0.0001 |
| HU | NU | 0.9 | -2.5 | < 0.0001 |
| MA | NU | 0.9 | 7.9 | < 0.0001 |

**Supplementary Table 3.** Point estimates and p-values for ΔO_x,A-B_, the difference, in days, between O_x, Institution A_ and O_x, Institution B_. P-values were generated via the student’s two-sample t test and corrected via the Benjamini-Hochberg method. BU, Boston University. HU, Harvard University. MA, Massachusetts. NU, Northeastern University.

| **Affiliation** | **B_0_** | **B_0_ 95% CI** | **B_1_** | **B_1_ 95% CI** | **McFadden’s R^2^** | **Overdispersion ratio** |
| --- | --- | --- | --- | --- | --- | --- |
| BU students | -8758.00 | (-10736.71,  -10736.76)* | 0.46 | (0.37, 0.57)* | 0.47 | 0.73 |
| BU employees | -9820.99 | (-13266.13,  -7015.17)* | 0.52 | (0.37, 0.70)* | 0.51 | 0.69 |
| MA | -5622.16 | (-5769.38,  -5478.22)* | 0.29 | (0.29, 0.30)* | 0.56 | 0.56 |

**Supplementary Table 4**. Logistic regression point estimates and 95% confidence intervals (CI) for intercept (B_0_) and slope (B_1_) parameters for students and for employees at Boston University (BU). * signifies that the 95% confidence interval does not include 0. MA, Massachusetts.

| **Affiliation A** | **Affiliation B** | **x** | **ΔO_x,A-B_ (days)** | **P-value** |
| --- | --- | --- | --- | --- |
| BU employees | BU students | 0.1 | -2.8 | < 0.0001 |
| BU employees | MA | 0.1 | 0.2 | 0.38 |
| BU students | MA | 0.1 | 3.0 | < 0.0001 |
| BU employees | BU students | 0.5 | -2.3 | < 0.0001 |
| BU employees | MA | 0.5 | 3.4 | < 0.0001 |
| BU students | MA | 0.5 | 5.7 | < 0.0001 |
| BU employees | BU students | 0.9 | -1.8 | < 0.0001 |
| BU employees | MA | 0.9 | 6.6 | < 0.0001 |
| BU students | MA | 0.9 | 8.3 | < 0.0001 |

**Supplementary Table 5.** Point estimates and p-values for ΔO_x,A-B_, the difference, in days, between O_x, Affiliation A_ and O_x, Affiliation B_. P-values were generated via the student’s two-sample t test and corrected via the Benjamini-Hochberg method. BU, Boston University. MA, Massachusetts.


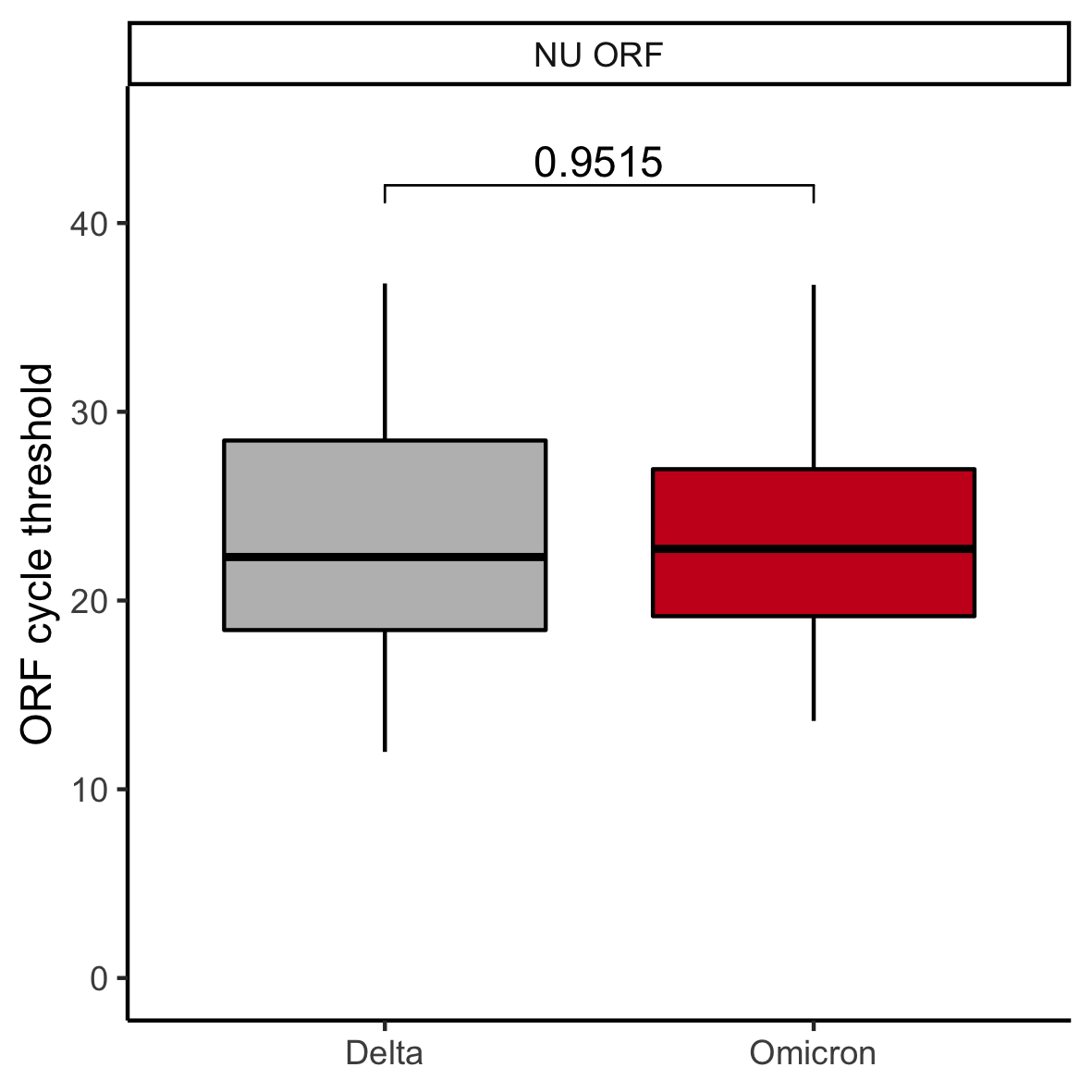


**Supplementary Figure 2**. ORF1ab cycle threshold (Ct) for Delta *vs.* Omicron cases at NU. Gray, Delta. Red, Omicron. The first, second, and third quartiles are within the box, with the median line bolded. The whisker length is 1.5 times the interquartile range (IQR), unless the furthest point is less than 1.5*(IQR) from the quartile. Outliers are displayed as points. P-value via Wilcoxon rank sum test and corrected via Benjamini-Hochberg method (across the 4 comparisons of **Figure 3** and the 1 comparison of **Supplementary Figure 2**).

| **Institution** | **Variant** | **Target** | **Median** | **Mean** |
| --- | --- | --- | --- | --- |
| BU | Delta | N1 | 21.6 | 22.9 |
| BU | Omicron | N1 | 25.1 | 25.2 |
| BU | Delta | N2 | 21.3 | 22.9 |
| BU | Omicron | N2 | 24.7 | 24.9 |
| HU | Delta | N1 | 24.3 | 24.6 |
| HU | Omicron | N1 | 28.0 | 27.7 |
| NU | Delta | N2 | 23.1 | 23.9 |
| NU | Omicron | N2 | 23.4 | 23.6 |
| NU | Delta | ORF | 22.3 | 23.4 |
| NU | Omicron | ORF | 22.7 | 23.1 |

**Supplementary Table 6**. Mean and median cycle threshold (Ct) values, per institution and per variant. BU, Boston University. HU, Harvard University. NU, Northeastern University.


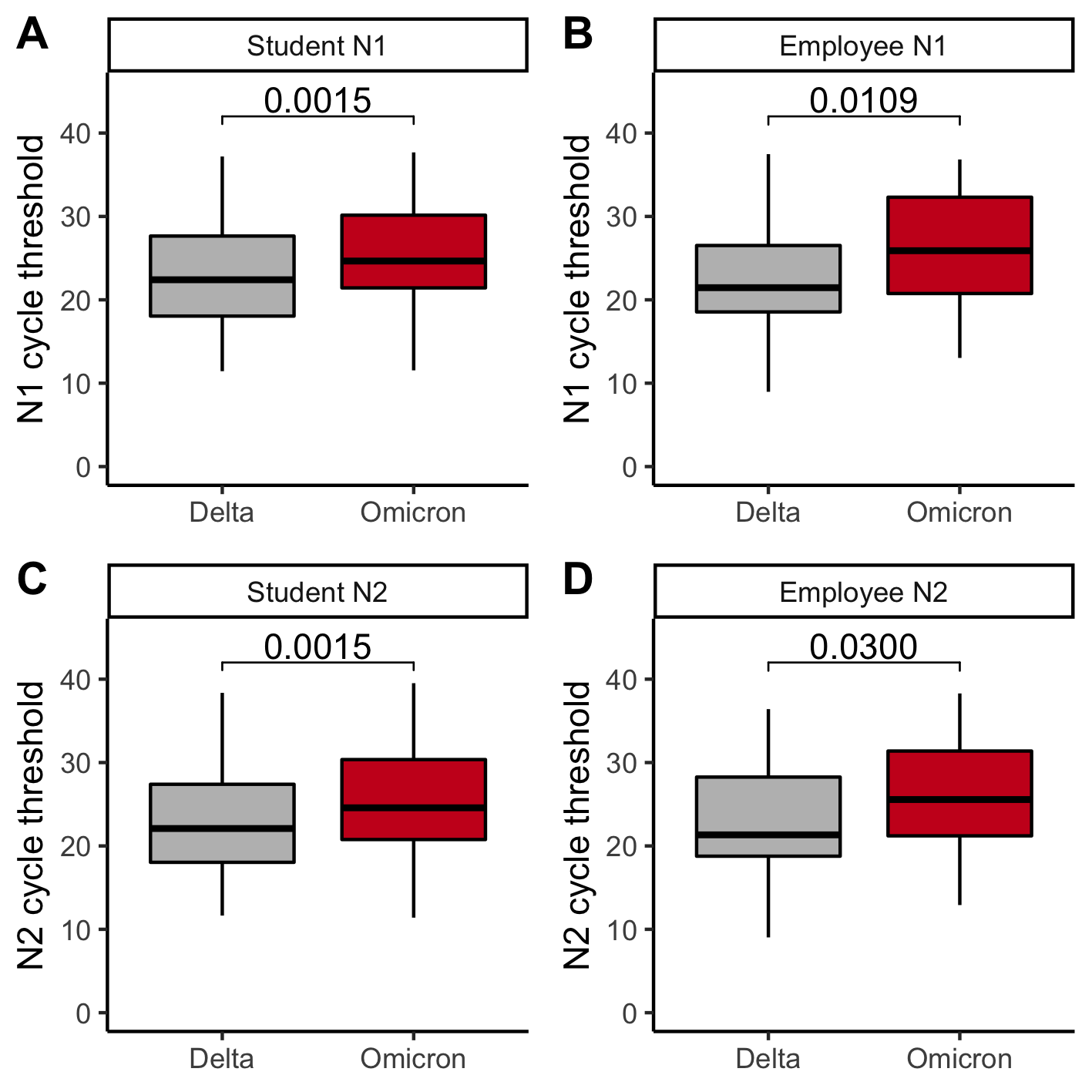


**Supplementary Figure 3. AB.** N1 cycle threshold for Delta *vs.* Omicron cases among BU students (**A**) and employees (**B**). **CD.** N2 cycle threshold for Delta *vs.* Omicron cases among BU’s students (**C**) and employees (**D**). **ABCD.** Gray, Delta. Red, Omicron. The first, second, and third quartiles are within the box, with the median line bolded. The whisker length is 1.5 times the interquartile range (IQR), unless the furthest point is less than 1.5*(IQR) from the quartile. Outliers are displayed as points. P-values via Wilcoxon rank sum test and corrected via Benjamini-Hochberg method (across the 4 comparisons of **Supplementary Figure 3**). BU, Boston University.

| **Affiliation** | **Variant** | **Primer** | **Median** | **Mean** |
| --- | --- | --- | --- | --- |
| BU employees | Delta | N1 | 21.5 | 22.9 |
| BU employees | Omicron | N1 | 25.9 | 26.0 |
| BU employees | Delta | N2 | 21.3 | 23.1 |
| BU employees | Omicron | N2 | 25.6 | 25.6 |
| BU students | Delta | N1 | 22.4 | 23.1 |
| BU students | Omicron | N1 | 24.7 | 25.4 |
| BU students | Delta | N2 | 22.1 | 22.9 |
| BU students | Omicron | N2 | 24.6 | 25.3 |

**Supplementary Table 7**. Mean and median cycle threshold (Ct) values at BU, per affiliation and per variant. BU, Boston University.

**References**

1. [Hamer DH, White LF, Jenkins HE, et al. Assessment of a COVID-19 Control Plan on an Urban University Campus During a Second Wave of the Pandemic. JAMA Netw Open **2021**; 4:e2116425.](http://paperpile.com/b/xPLGNk/ekA2Q)

2. [Bouton TC, Lodi S, Turcinovic J, et al. Coronavirus Disease 2019 Vaccine Impact on Rates of Severe Acute Respiratory Syndrome Coronavirus 2 Cases and Postvaccination Strain Sequences Among Health Care Workers at an Urban Academic Medical Center: A Prospective Cohort Study. Open Forum Infect Dis **2021**; 8:ofab465.](http://paperpile.com/b/xPLGNk/FRBJn)

3. [EMERGENCY USE AUTHORIZATION (EUA) SUMMARY Quaeris SARS-CoV-2 Assay. 2021; Available at:](http://paperpile.com/b/xPLGNk/Q4Njl) <https://www.fda.gov/media/149445/download>[.](http://paperpile.com/b/xPLGNk/Q4Njl)

4. [Welch NL, Zhu M, Hua C, et al. Multiplexed CRISPR-based microfluidic platform for clinical testing of respiratory viruses and SARS-CoV-2 variants. bioRxiv. 2021; Available at:](http://paperpile.com/b/xPLGNk/gjHvO) <http://medrxiv.org/lookup/doi/10.1101/2021.12.14.21267689>[.](http://paperpile.com/b/xPLGNk/gjHvO)

5. [Granato PA, Kimball SR, Alkins BR, Cross DC, Unz MM. Comparative Evaluation of the Thermo Fisher TaqPath TM COVID-19 Combo Kit with the Cepheid Xpert® Xpress SARS-CoV-2 Assay for Detecting SARS-CoV-2 in Nasopharyngeal Specimens. **2021**; Available at:](http://paperpile.com/b/xPLGNk/ivKEu) <https://www.researchsquare.com/article/rs-429726/latest.pdf>[.](http://paperpile.com/b/xPLGNk/ivKEu)

6. [CDC Case Task Force. United States COVID-19 cases and deaths by state over time. 2020; Available at:](http://paperpile.com/b/xPLGNk/sh04M) <https://data.cdc.gov/Case-Surveillance/United-States-COVID-19-Cases-and-Deaths-by-State-o/9mfq-cb36>[. Accessed 21 January 2022.](http://paperpile.com/b/xPLGNk/sh04M)

7. [Shu Y, McCauley J. GISAID: Global initiative on sharing all influenza data - from vision to reality. Euro Surveill **2017**; 22. Available at:](http://paperpile.com/b/xPLGNk/fEMXW) <http://dx.doi.org/10.2807/1560-7917.ES.2017.22.13.30494>[.](http://paperpile.com/b/xPLGNk/fEMXW)

8. [Elbe S, Buckland-Merrett G. Data, disease and diplomacy: GISAID’s innovative contribution to global health. Glob Chall **2017**; 1:33–46.](http://paperpile.com/b/xPLGNk/34pJx)

9. [Khare S, Gurry C, Freitas L, et al. GISAID’s Role in Pandemic Response. China CDC Wkly **2021**; 3:1049–1051.](http://paperpile.com/b/xPLGNk/j505P)

10. [Payne EH, Gebregziabher M, Hardin JW, Ramakrishnan V, Egede LE. An empirical approach to determine a threshold for assessing overdispersion in Poisson and negative binomial models for count data. Commun Stat Simul Comput **2018**; 47:1722–1738.](http://paperpile.com/b/xPLGNk/zeP8M)

11. [McCullagh P, Nelder JA. Generalized linear models. Routledge, 2019.](http://paperpile.com/b/xPLGNk/zSjAH)

12. [McFadden D. Regression-based specification tests for the multinomial logit model. J Econom **1987**; 34:63–82.](http://paperpile.com/b/xPLGNk/1t9cj)

13. [McFadden D. Conditional logit analysis of qualitative choice behavior. In: Zarembka P, ed. Frontiers in Econometrics. Academic Press, New York, 1973: 105–142.](http://paperpile.com/b/xPLGNk/gbo4t)
